# Socio-Demographic Influences on Dietary Habits and Nutritional Awareness: A Case Study of Polish Biathlon Association National Team Members

**DOI:** 10.3390/nu16213784

**Published:** 2024-11-04

**Authors:** Agnieszka Górka-Chowaniec, Magdalena Niewczas-Dobrowolska, Anna Akbaş, Eduard Bezuglov, Tadeusz Sikora, Zbigniew Waśkiewicz

**Affiliations:** 1 Sport and Tourism Management Faculty, Jerzy Kukuczka Academy of Physical Education, 40-065 Katowice, Poland; gorkachowaniec@interia.eu; 2 Institute of Quality Sciences and Product Management, Cracow University of Economics, 31-510 Cracow, Poland; niewczam@uek.krakow.pl; 3 Institute of Sport Science, Jerzy Kukuczka Academy of Physical Education, 40-065 Katowice, Poland; a.akbas@awf.katowice.pl; 4 Department of Sports Medicine and Medical Rehabilitation, Sechenov First Moscow State Medical University, 119435 Moscow, Russia; e.n.bezuglov@gmail.com; 5 Department of Quality Management, Cracow University of Economics, 31-510 Cracow, Poland; sikorat@uek.krakow.pl

**Keywords:** dietary habits, nutritional awareness, socio-demographic factors, professional athletes

## Abstract

Introduction: This study investigated the influence of sociodemographic factors on the dietary habits of athletes of the Polish Biathlon Association. Focusing on age, education, employment status, and gender, this research assesses food choices, meal preparation, and nutritional awareness within a structured sports environment. A cross-sectional survey of 54 athletes was conducted using a modified “Eating Habits of Poles” questionnaire to explore food selection, preparation methods, consumption patterns, and nutritional perspectives. This focus on biathletes emphasizes their distinct dietary needs, which arise from the demanding combination of endurance and precision in their sport, providing valuable insights for tailored dietary strategies to enhance their performance and overall health. Results: The results indicate that age, education, and employment status significantly influence Polish biathletes’ dietary habits and nutritional awareness. Older athletes (under 23 years) demonstrated significantly higher nutritional awareness regarding modern dietary trends (*p* = 0.015). In contrast, 50% of higher-education athletes were more engaged in meal planning and healthier food choices than those with elementary education (*p* = 0.031). Employment status also played a role; 70% of the athletes were students who exhibited more convenience-based food choices, whereas 30% were employed and maintained more structured eating patterns (*p* = 0.008). Minimal gender differences were found, with 50% of male and 50% of female athletes showing similar dietary habits, likely due to standardized nutrition programs provided to all athletes. Conclusions: This indicates a potential need for further research to determine whether professional dietary support can effectively address typical gender-related variations in food behavior and lead to improvements in dietary outcomes. This study highlights the importance of targeted nutrition education and professional support for optimizing the nutritional habits of professional athletes. This emphasizes that socio-demographic factors such as age, education, and employment status significantly shape these behaviors, underscoring the need for personalized nutritional strategies within athletic programs.

## 1. Introduction

Athletes’ diets, shaped by their daily choices, are pivotal in influencing their preparation, performance, and recovery after competitions. Optimal nutrition is essential not only for supporting physical activity and enhancing sports performance [1,2,3,4] but also for minimizing the risk of injuries [5] and speeding up recovery after intense exertion [6,7]. Athletes are expected to adhere to well-structured eating habits, ensuring their food intake meets the necessary quantity and quality for high-level performance.

Athletes form a group with specific nutritional needs, and a well-balanced diet is critical for maintaining their health and exercise performance. Eating behaviors are shaped by various individual and environmental factors [8]. Research has shown that athletes’ dietary behaviors are dynamic and influenced by multiple factors [9,10,11,12,13]. Despite expectations, studies have uncovered certain irregularities in the eating habits of athletes [14,15,16,17,18].

These behaviors are influenced by an integrated and interacting system of factors, including genetic predispositions, hormonal regulation, sensory development, and environmental and cultural contexts. These influences can be grouped into biological traits, demographic characteristics, and socioeconomic factors such as age, gender, family size, and income level. Studies have frequently highlighted the importance of sociodemographic factors in shaping individual eating habits [19,20,21]. 

Age, sex, socioeconomic status (SES), nutritional knowledge, and cultural influences are critical determinants of dietary habits among athletes. Younger athletes and women often exhibit different nutritional patterns than older athletes and men, with gender differences evident in meal preparation and fast-food consumption [22,23]. SES impacts access to nutritious foods and knowledge, influencing athletes’ dietary practices [24,25,26]. Access to resources like sports dietitians also significantly enhances nutritional practices, particularly for less experienced athletes [11,24]. Moreover, cultural norms and food environments shape athletes’ dietary choices, emphasizing the importance of tailored nutritional interventions [14] 

Education is a critical determinant among significant factors, with more educated individuals generally displaying excellent nutritional knowledge and healthier eating behaviors. Research has consistently shown a positive correlation between nutritional awareness and the adoption of health-promoting dietary habits, although the strength of this correlation can vary [10,27,28]. Additionally, economic status is crucial in shaping dietary habits, influencing food quality and the frequency of nutrient deficiencies [24,25,26]. Furthermore, athletes’ competitive environments and emotional states may challenge their ability to follow optimal nutritional guidelines [11]. While many factors affecting the general population also apply to athletes [11,29,30,31,32,33], the athletic environment introduces specific challenges related to performance, such as the influence of coaches and peers and competition-related stress [10,27,28,34,35,36]. These stimuli and attitudes toward food can significantly impact dietary choices. As the literature reveals, socio-demographic factors influence dietary habits in sports settings [1,2,6]. However, more research should focus explicitly on biathletes, suggesting a need for further exploration [9,36,37,38,39,40,41].

This research builds on previous studies by focusing on a relatively underexplored group—professional biathletes—whose dietary habits differ significantly from those of athletes in other disciplines due to the unique combination of endurance and precision required in their sport. Unlike previous works broadly examining athletes’ nutrition across different sports, this study examines how sociodemographic factors such as age, education, and employment status influence biathletes’ food choices and nutritional awareness. By doing so, we aim to contribute to more tailored and effective dietary strategies that support the distinct demands of biathletes, providing new insights that extend beyond the scope of the existing literature.

Understanding the dietary behaviors of athletes, particularly within structured sports environments, can provide valuable insights for designing interventions to promote optimal health and performance. This study, therefore, sought to analyze how critical socio-demographic factors, such as age, education, employment status, household size, and region of residence, influence the dietary habits of athletes from the Polish Biathlon Association. Specifically, it aimed to explore how these variables affect food choices, preparation, consumption, and athletes’ attitudes toward nutrition to identify the critical determinants of their dietary behavior.

After defining the aims of the study, several objectives were established to guide the investigation. The first aim was to examine how age and education influence athletes’ dietary habits and nutritional awareness within the context of Polish biathlon. We hypothesized that younger and higher-educated athletes would demonstrate a more significant understanding of modern dietary trends. The second aim was to explore the impact of employment status on food choice and meal planning among athletes in a structured sports environment. Additionally, this study sought to investigate whether gender differences influence dietary habits when standardized nutritional programs are implemented among professional athletes.

## 2. Materials and Methods

### 2.1. Study Design and Setting

The adopted cross-sectional research design used the original “Eating Habits of Poles” research tool with a descriptive phenomenological approach to collect qualitative data in naturalistic conditions. The demographic characteristics section (the third part of the questionnaire) was adapted in agreement with the Polish Biathlon Association (PZB) management to suit the specific group, the PZB Team. The modifications concerned the demographic characteristics of the studied population, such as the age of respondents (junior under 23 years old and senior over 23 years old), employment status (student, unemployed), number of household members (1–3 family members and more than four members), and place of residence. Due to the personal declaration of residence (province), it was decided to retain three of the 16 provinces in Poland (Silesian, Lower Silesian, and Lesser Poland).

Before proceeding with the primary research, pilot studies were conducted using a research method based on in-depth individual interviews involving a target group of 20 respondents—athletes. The primary research examining the influence of sociodemographic factors on the eating habits of the Polish Biathlon Association’s team by sport level (juniors vs. seniors) was conducted between March and June 2022. The research instrument used at this stage of the study was an original questionnaire that included the characteristics of the respondents and 35 main questions classified into four areas of analysis (research constructs): selection of food products based on their consumption type (SP), preparation and production of food (EP), methods of consuming food (EF), and opinions on food and nutrition (VF). The respondents’ task was to assess the extent to which they agreed with the statements regarding their eating habits, which were grouped into four areas. The respondents’ evaluations, reflecting the degree of acceptance of a given phenomenon or opinion, was conducted using a 5-point Likert scale, ranging from 1 (strongly disagree) to 5 (strongly agree). The original assumptions of the research process aimed to reach respondents through indirect and direct channels using the same research tool, namely a survey: “Eating Habits of the Staff of the Polish Biathlon Association”. However, considering the period of intense sports activity that the team was experiencing and the athletes’ travel schedule, the author abandoned one of the planned initial data collection methods (face-to-face interviews) and opted to conduct the study using the CAWI method.

### 2.2. Study Population and Sampling Procedure

This study was conducted in Poland among the National Team of the Polish Biathlon Association population for the 2022/2023 season. It involved 100% of the population of athletes from the Regulatory Team of the Polish Biathlon Association for the 2022/2023 season, divided into specific subsections according to age range: Senior Women’s Team, Senior Men’s Team, Junior Women’s Team, and Junior Men’s team. The study sample comprised 61 athletes. The response rate was 54.

### 2.3. Data Collection and Quality Assurance

The guide for computer-assisted online interviews was prepared by one of the authors of this study and the team involved in the research. The guide was comprehensive and covered all aspects of the team’s goals. He encouraged participants to share their experiences in their own words and in their way, without forcing them into categories or classifications imposed by the interviewer. The tool was prepared in Poland. The interview guide was preliminarily tested and refined to improve its clarity.

### 2.4. Data Management and Analysis

The Shapiro–Wilk test was used to check for normal distribution, and variance homogeneity was assessed using Levene’s test. Since the assumption of normality was not met, we applied the non-parametric Mann–Whitney U test to examine the influence of sex, age, employment, and number of household members on the total score (summed across all questions) for each of the four questionnaire categories (SP, FP, EF, VF). Additionally, the non-parametric Kruskal–Wallis test was employed to evaluate the effects of education and voivodship on the total score within each category.

In the second analysis, we focused on individual responses within each category. Mann–Whitney U and Kruskal–Wallis tests were used to compare responses to personal questions (Likert scale 1–5) within the four categories across different demographic and socioeconomic groups. These tests were chosen owing to the ordinal nature of the data.

Effect sizes were reported as r-values for the Mann–Whitney U test, with thresholds for small (0.1), medium (0.3), and large effects (0.5). For the Kruskal–Wallis test, partial eta-squared (η^2^) values were reported, with thresholds for small (0.01), medium (0.06), and large effects (0.14).

All statistical analyses were performed using Statistica v.13.3 (TIBCO Software Inc.). The results are presented as the mean ± standard deviation. The alpha level was set at 0.05.

### 2.5. Ethics Approval and Consent to Participate

This study was approved by the Bioethical Committee of the Academy of Physical Education in Katowice (KB/54/2021). All procedures in this study were conducted in accordance with the Declaration of Helsinki, ensuring the complete anonymity and confidentiality of all participants. Personal and sensitive data were thoroughly safeguarded, and the collected data could not be used to identify individuals or personal cases. Since the study used online surveys, completing the survey was considered as informed consent, eliminating the need for a separate consent form. The participants were fully informed of the study objectives and retained the right to withdraw at any time. No personal identifiers, such as names, were disclosed.

## 3. Results

### 3.1. Characteristics of Participants

Table 1 presents the demographic and socioeconomic characteristics of the Polish athletes who participated in the study. These characteristics were described by sociodemographic factors, such as sex, age, level of education, employment, number of households, and voivodship. The initial sample used in the study of the Polish Biathlon Team’s dietary habits consisted of 54 respondents (61 team members in the 2022/2023 season). Table 1 presents the preliminary demographic characteristics of the respondents along with their division into individual consumer segments. The athletes participating in the study were representatives of three provinces in Poland. Among the 54 complete responses, 27 (50%) were from women, and 27 (50%) were from men. Most respondents were under 23 years of age (junior category), representing nearly 75% of the sample, with 38 individuals (70.37%) falling within this age group. The next group consisted of respondents who presented a family model with to 1–3 members (*n* = 22; 40.74% of the surveyed). A significant share was held by respondents who created family models with four or more household members (*n* = 32; 59.25% of respondents). As the data in Table 1 indicate, students were predominant among the surveyed individuals (*n* = 38, 70.37%). The remaining group comprised unemployed individuals (*n* = 16, 29.63%). Almost 1/4 of the respondents (*n* = 13, 24.07%) declared having a primary education. A slightly larger percentage was comprised of individuals with higher education (*n* = 14, 25.93%). The most significant percentage of respondents was athletes with higher education (*n* = 27, 50%). These constituted half of the respondents.

### 3.2. Questionnaire

The study was conducted using a modified questionnaire, “Eating Habits of Poles”, (Table 2), which aims to examine the lifestyles of consumers and their food purchasing decisions. In developing the original research tool, “Eating Habits of Poles”, the methodological assumptions were based on several established models and methods. These included: Khan’s food preference model [41], FRL model [42,43], customer retention model [44], RPB model [45], DINESERV method [46,47], SERVQUAL method [48], and Ecoserv method [49].

The “PZB Eating Habits Questionnaire” measures 69 questions regarding food product choices, preferred meal preparation methods, food consumption methods, and opinions about food and nutrition. Consumers typically rated these items on a 5-point Likert scale (from 1 (“strongly disagree”) to 5 (“strongly agree”)). The questionnaire used in this study encompassed 35 dimensions across four interconnected areas: food product selection, food preparation and production, methods of food consumption, and perspectives on food and nutrition. This research tool can be used widely in empirical markets. The questionnaire can be used for consumer segmentation, market segmentation, or for studying the eating habits of consumers in general or of a specific type of consumer.

In this research, the modified “The Eating Habits of Poles” questionnaire was used to examine the food behavior of the Polish Biathlon Association Team. A modified version of the questionnaire was administered. It was based on the original “The Eating Habits of the Poles” questionnaire. “The Eating Habits of Poles” questionnaire implemented in this study consists of five domains: food purchase, food preparation, food consumption, and food opinions/attitudes. There were 35 statements (items) to be assessed by consumers using a 5-point scale (from 1 (“completely disagree”) to 5 (“completely agree”)).

This questionnaire was tested for reliability in a study of biathletes. Cronbach’s alpha coefficient was calculated. Its value ranges from 0 to 1. A high Cronbach’s alpha value indicates that the response values for each participant across a set of questions were consistent.

The accepted Cronbach’s alpha coefficient value is ≥0.7 or more (maximum of 1). In this research, the Cronbach’s alpha value was 0.8287; therefore, this questionnaire is consistent and can be used to examine food-related lifestyles among the National Biathlon Team.

In the first domain, “food purchase”, the aspects of food labeling and choice, information consumers use to choose food products, and preferred places to buy food are rated. In the second domain, “food preparation”, respondents were asked how they planned and prepared meals. In the third domain, “food consumption”, participants rated their preferences regarding how they want to consume food. In the last domain, “food opinion,” participants expressed their views about the characteristics of food and diets that were important to them.

### 3.3. The Influence of Demographic and Socioeconomic Factors on Nutritional Habits in Polish Biathletes—A Total Score Analysis

There was no significant effect of sex, number of household members, or voivodship on any of the four questionnaire categories (*p* > 0.05). However, a significant main effect of age was found in the VF category (food opinion), indicating significantly higher scores in the older group (more than 23 years old) compared to those aged 23 years or less. Additionally, a significant difference was found in the type of employment in the SP category (food purchase), with higher scores among students than among employed individuals (Table 3).

The type of *education* significantly impacted the food preparation (FP), food consumption (EF), and food opinion (VF) categories, with post hoc analysis revealing considerably higher scores among individuals with higher education than among those with elementary education (FP: *p* = 0.026; EF: *p* = 0.021; VF: *p* = 0.01) (Table 3). The effects of *age* on food opinion (VF), *employment* on food purchase (SP), and *education* on food preparation (FP), food consumption (EF), and food opinion (VF) were further explored by considering the responses to individual questions within those categories.

### 3.4. The Influence of Demographic and Socioeconomic Factors on Nutritional Habits in Polish Biathletes—An Analysis of Individual Questions

#### 3.4.1. Effect of Age

**Figure 1:**
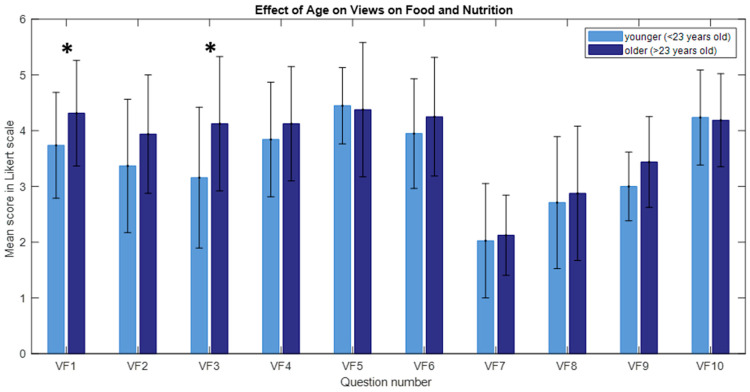
The effect of age on views on food and nutrition (VF). The bars represent the mean scores, while the error bars indicate the standard deviations (* *p* < 0.05).

The U Mann–Whitney test revealed significantly higher scores in responses to questions VF1 (“I am characterized by great nutritional awareness”) (U = 197.5, n_1_ = 16, n_2_ = 38, ***p*** = 0.044, r = 0.27) and VF3 (“Because of the problems I have discovered, I am trying to limit the meat and meat product I buy in stores”) (U = 182.5, n_1_ = 16, n_2_= 38, *p* = 0.022, r = 0.31) among older individuals (Figure 1). This suggests that those aged 23 years and older were more likely to be interested in food products and an active approach to food quality and safety issues. The older the biathlete, the higher their food awareness. Older respondents ranked higher in the VF domain (food opinions) (Figure 1). There was no significant impact of sex on the answers, as the biathletes had the same diet specialists responsible for their diets. For this reason, they have a limited ability to decide on the food they consume.

#### 3.4.2. Effect of Education

**Figure 2:**
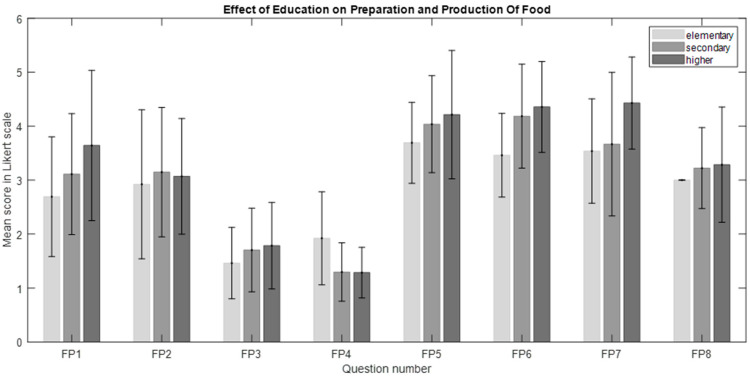
The effect of education on preparation and production of food (FP). The bars represent the mean scores, while the error bars indicate the standard deviations.

**Figure 3:**
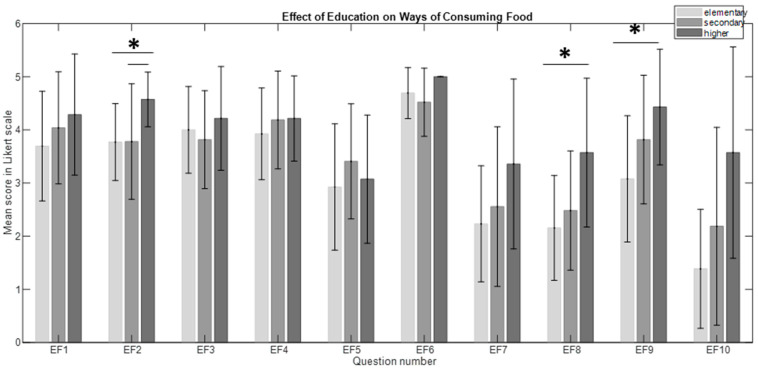
The effect of education on ways of consuming food (EF). The bars represent the mean scores, while the error bars indicate the standard deviations (* *p* < 0.05).

The Kruskal–Wallis test revealed a significant main effect of education on the “Preparation and Production of Food” category in questions FP4, FP6, and FP7 (H_(2, 54)_ = 7.047, *p* = 0.03, η^2^ = 0.1). However, post hoc analysis did not show any specific differences between the groups with different education levels (Figure 2). The significant main effects of education on the “Ways of Consuming Food” category were found in questions EF2 (H_(2, 54)_ = 8.87, *p* = 0.012, η^2^ = 0.13), EF6 (H_(2, 54)_ = 7.62, *p* = 0.022, η^2^ = 0.11), EF8 (H_(2, 54)_ = 8.83, *p* = 0.012, η^2^ = 0.13), EF9 (H_(2, 54)_ = 9.7, *p* = 0.008, η^2^ = 0.15), and EF10 (H_(2, 54)_ = 8.5, *p* = 0.014, η^2^ = 0.13). Post hoc analysis revealed particular differences between those with higher versus secondary education (*p* = 0.049) and higher versus elementary education (*p* = 0.043) in EF2, which may suggest that the higher the level of education, the higher the involvement in the degree of food processing and food awareness. Additionally, there were significant differences between higher- versus elementary-educated individuals in EF8 and EF10 (see Figure 3), which may indicate that consumers with higher education levels pay more attention to how they consume food and the food they consume, such as their nutritional value.

**Figure 4:**
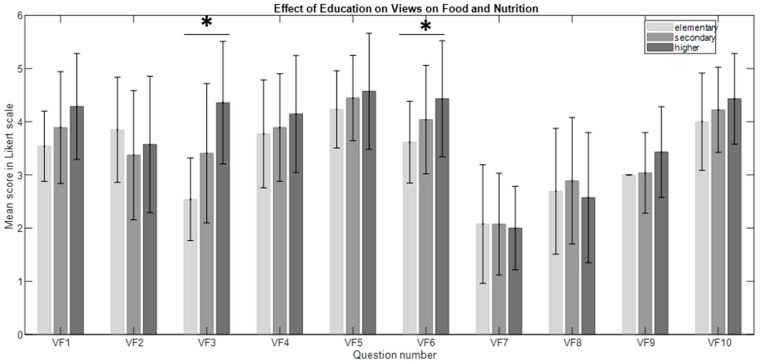
The effect of education on views on food and nutrition (VF). The bars represent the mean scores, while the error bars indicate the standard deviations (* *p* < 0.05).

The significant main effects of education on the “Views on Food and Nutrition” category were found in questions VF3 (H_(2, 54)_ = 12.82, *p* = 0.002, η^2^ = 0.21) and VF6 (H_(2, 54)_ = 7.71, *p* = 0.021, η^2^ = 0.11). Post hoc analysis revealed differences between those with higher versus elementary education in both VF3 (*p*= 0.002) and VF6 (*p* = 0.023) (see Figure 4). This also shows a higher awareness of food and the influence of food on our health and well-being. Moreover, they showed greater engagement in food choices and food ingredients.

#### 3.4.3. Effect of Employment

**Figure 5:**
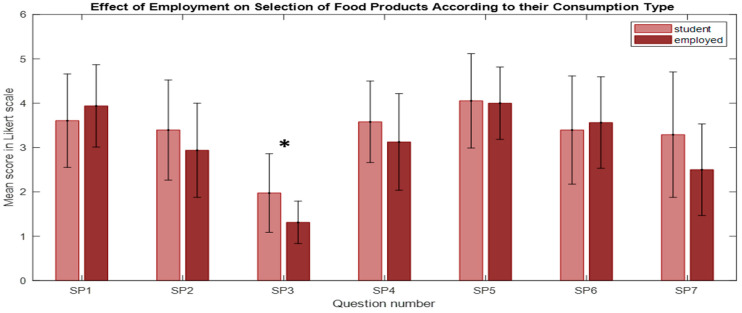
The effect of employment on the selection of food products according to their consumption type (SP). The bars represent the mean scores, while the error bars indicate the standard deviations (* *p* < 0.05).

The U Mann–Whitney test revealed significantly higher scores in responses to question SP3 (“I do not attach much importance to buying food products, I usually buy what is ‘at hand’”) (U = 171, n_1_ = 16, n_2_ = 38, *p* = 0.012, r = −0.34) among students when compared to employed individuals (Figure 5). This suggests that students were more likely to agree with this statement. This also corresponds to education level and age. Higher involvement in food purchase and food preparation comes with education level and age.

As in the group of general consumers, this research among biathletes showed that age influenced awareness about food’s nutritional value and taking an active approach towards the product they consume. A similar situation occurs in the case of the respondents’ education level. The higher the education, the greater the awareness and interest in food issues and the active approach towards food, diets, ingredients, and food processing. Usually, in consumer food studies, sex plays an essential role in influencing the answers. In the present study, no impact of sex was observed. This may indicate that this activity plays a central role in professional sports activities, not sex. A more extended period of involvement in professional sports activities (as well as a higher level of education) results in a greater interest in food and nutrition issues and shapes attitudes towards food.

## 4. Discussion

This study aimed to explore the sociodemographic determinants of dietary habits among athletes of the Polish Biathlon Association. The findings provide insights into how age, education, employment status, gender, and household structure shape professional athletes’ food choices, meal preparation, and nutritional awareness. Understanding these relationships is crucial because proper nutrition is key to athletic performance, recovery, and overall health. In a highly controlled environment such as professional sports, it is essential to identify the specific influences that lead to variations in dietary behavior. The results highlight both expected and unexpected patterns, discussed in detail in the following sections, each addressing a core area of influence on athletes’ dietary habits.

### 4.1. Age and Nutritional Awareness

This study found that older athletes (<23 years) exhibited higher scores in food awareness and nutritional consciousness than their younger counterparts, particularly in the “Views on Food and Nutrition” category. According to their experience, older athletes proactively engaged with modern dietary trends, particularly meat consumption. These findings do not align with the existing literature, which suggests that age alone does not directly affect nutritional knowledge. Taye et al. [50] and Kathure et al. [51] found no significant differences in nutrition knowledge across age groups. However, research by Murathan [52] indicated that older athletes tend to have more positive attitudes toward nutrition, which is essential for maintaining a balanced diet.

Furthermore, Staśkiewicz et al. [53] observed that athletes with higher nutritional awareness demonstrated better dietary practices irrespective of age, which can positively influence performance. The role of coaches and nutritionists is paramount in enhancing nutritional knowledge. Athletes often rely on informal sources of information, which can lead to suboptimal food choices [52]. This emphasizes the need for professional guidance in nutrition, particularly for athletes [54].

### 4.2. Education as a Major Determinant of Food Behavior

The study revealed that higher-education athletes scored significantly higher on food preparation, consumption, and food opinions than those with lower educational attainment. Higher education has been linked to greater food awareness, involvement in meal planning, and healthier food choices. These results are consistent with the findings of broader research. Higher educational attainment has been shown to correlate with healthier dietary patterns, including increased consumption of fiber, vitamins, and antioxidants [55,56]. In the Tromsø Study, Jacobsen and Nilsen [57] reported that educated individuals had lower fat and higher dietary fiber, which is particularly beneficial for athletes. Furthermore, socioeconomic factors associated with higher education, such as better access to food and more significant cultural capital, influence food choices [58,59].

### 4.3. Employment Status and Spontaneity in Food Choices

Athletes who were students exhibited more spontaneous food choices, often driven by convenience rather than careful planning, compared to their employed counterparts. This pattern is likely influenced by time constraints and financial limitations student-athletes face. Previous research corroborates these findings by showing that employment status significantly affects dietary habits. Caban-Martinez et al. [60] found that employed individuals tend to have more structured meal patterns, whereas students or unemployed individuals exhibit more erratic eating behaviors. Additionally, social influences in the workplace can shape food choices, with employed athletes potentially benefiting from healthier dietary norms [61]. The intersection of employment, education, and access to nutritional resources further supports the need for targeted interventions for student-athletes [62].

### 4.4. Professional Sports and Gender Differences

This study found no significant differences in dietary habits between male and female athletes, which may be attributed to the structured dietary programs provided by professional nutritionists. This contrasts with trends in the general population, where sex often plays a crucial role in nutritional choices due to cultural and social factors. This finding is supported by the literature showing that professional support can neutralize gender-based differences in food behavior [63,64]. However, female athletes often face unique nutritional challenges, including body image concerns and physiological fluctuations, which require tailored interventions [65,66]. The lack of gender differences observed in this study suggests that professional guidance may mitigate these issues in elite sports settings.

### 4.5. Limited Influence of Household Size and Region

Household size and region of residence did not significantly influence athletes’ dietary habits, likely because of the standardized nature of their sports environment, which overrides personal or regional differences. While this finding may seem surprising, it aligns with research suggesting that income and education play a more decisive role in food choices than household size or region [67]. Cohen and Babey [68] argued that external food environments, marketing, and individual attitudes toward health are often more robust determinants of dietary behavior than demographic variables. These findings highlight that professional sports are controlled and uniform, and that standardized programs guide athletes’ food choices.

## 5. Conclusions

Age and education significantly influence dietary habits. Older athletes showed greater nutritional awareness, particularly regarding adopting modern dietary trends. Higher education was correlated with healthier food choices and greater involvement in meal planning, underscoring the importance of nutrition education for performance. Employment status affects food choices. Due to time and financial constraints, student-athletes exhibited more spontaneous, convenience-driven eating, whereas employed athletes had more structured eating habits. Tailored nutritional strategies are required for student-athletes. Due to standardized nutritional programs, sex differences in dietary habits were minimal, suggesting that professional support neutralizes typical gender-related variations. However, attention to the specific needs of female athletes is essential for their optimal health and performance.

## 6. Practical Recommendations for Coaches and Sports Nutritionists

1. Targeted Nutrition Education: Coaches and nutritionists should focus on providing targeted nutrition education to younger athletes and those with lower education levels to enhance awareness of modern dietary trends and promote healthier meal planning.
2. Support for Student-Athletes: Since student-athletes often make convenience-based food choices, practical time management and budget-friendly meal preparation workshops can help them make better dietary decisions within their constraints.
3. Standardized Nutritional Programs: Nutritionists should continue implementing standardized nutritional programs that minimize gender differences, ensuring equitable access to nutrition resources across all athletes.
4. Individualized Strategies: Given the role of sociodemographic factors, nutritionists are encouraged to develop individualized strategies that consider athletes’ age, education, and employment status to optimize nutritional outcomes and performance.

## 7. Contribution to Sports Nutrition

The study reinforces the importance of socio-demographic factors in shaping athletes’ dietary habits, emphasizing the value of personalized nutrition programs. By understanding specific needs based on age, education, and employment, sports nutritionists can design tailored interventions that enhance nutritional awareness and dietary behaviors, ultimately contributing to improved health and athletic performance. This targeted approach provides actionable insights for professional practice within structured sports environments.

## 8. Limitations of the Study

The cross-sectional design limits the ability to establish causal relationships between sociodemographic factors and dietary habits, thus restricting the findings to associations. Additionally, the relatively small sample size of 54 respondents may have reduced the generalizability of the results to broader athletic populations. The study also relied on self-reported data, which can introduce bias due to potential inaccuracies in participants’ recall or reporting of their dietary behaviors. A fundamental limitation of this study is the lack of a formal sample size calculation. Due to the study’s focus on the Polish Biathlon Association, the sample consisted of all 54 available athletes from the official national teams, surveyed using the CAWI (computer-assisted web interview) method. While this represents nearly the entirety of this specific group, it may limit the broader generalizability of the findings. Additionally, the absence of sample size estimation using statistical methods such as G*Power was due to the practical constraints of studying a fixed population. Future research could expand the sample to include biathletes beyond the national team to enhance generalizability and provide further validity to the findings. Furthermore, the relatively large standard deviations observed in categories such as food preparation, consumption, and nutritional awareness indicated substantial response variability. This variability suggests that the observed effects may not be uniformly distributed across all participants, potentially weakening the reliability and robustness of our findings.

## Figures and Tables

**Table 1:** Demographical characteristics of participants.

Variables		*n*	*%*
Sex			
Women		27	50
Men		27	50
Age			
Less than 23		38	70.37
23 and over		16	29.63
Education			
Elementary		13	24.07
Secondary		27	50
Higher		14	25.93
Employment			
Student		38	70.37
Employed		16	29.63
No. of household members			
1 to 3		22	40.74
4 and more		32	59.25
Voivodship			
Silesia		13	24.07
Lower Silesia		19	35.19
Lesser Poland		22	40.74

**Table 2:** “The Eating Habits of PZB” questionnaire used in this research.

Code	Food Product Selection
SP1	I read the information on the labels of various products and, based on this, I make choices about products that are beneficial to my health.
SP2	When purchasing food products, I take into account the opinions of other people (experts on the subject).
SP3	I don’t attach much importance to buying food products, I usually buy what is “at hand”.
SP4	I like to buy products from “health” food and organic food stores.
SP5	When I buy food products, I pay attention to the price.
SP6	Current promotions are important to me when making product purchasing choices.
SP7	Usually, I make the final decision on what to buy only in the store.
FP	Food preparation and production
FP1	I don’t like to spend too much time preparing and making meals.
FP2	My cooking process is influenced by my knowledge of the cuisines of other nations.
FP3	In our home, instead of cooking, we often use ready-made meals (dumplings, breaded meat, bigos, beans, ready-made sauces, soups, etc.).
FP4	I use a lot of instant products, e.g., ready-made baking mixes, soups, and powdered sauces.
FP5	Meals should be planned in advance.
FP6	In modern times, shopping and cooking should be shared equally between women and men.
FP7	When planning my menu, I take into account the seasonality of food products (i.e., in winter I eat more pickled/fermented foods and in summer I eat more tomatoes, radishes, cucumbers, etc.)
FP8	Having a small child, I try to prepare meals at home more often rather than eating out.
EF	Methods of food consumption
EF1	Eating with friends is a great way to spend your free time.
EF2	I pay great attention to ensuring that the products I buy are as minimally processed as possible.
EF3	I compare similar products to make sure the quality matches the price.
EF4	I like trying new foods that I have never eaten before.
EF5	I pay attention to ecological products for which I am willing to pay more.
EF6	I prefer fresh products over frozen ones.
EF7	Eating out is a regular part of my family’s eating habits.
EF8	I prefer to invite family (friends) to a restaurant than prepare a meal for them at home.
EF9	I eat 4–5 meals a day every 3–4 h.
EF10	I have been on a “boxed” diet at least once in my life.
VF	Views on food and nutrition
VF1	I am characterized by great nutritional awareness.
VF2	I know what the Healthy Eating Plate looks like, which includes the latest recommendations for healthy eating.
VF3	In view of the problems I have discovered, I am trying to limit the meat and meat product I buy in stores.
VF4	In my family home, a lot of attention is paid to eating meals together.
VF5	I believe that the obesity problem in modern society is due to the frequent consumption of fast food and highly processed foods.
VF6	Awareness of the existence of many diet-related diseases encourages me to pay more attention to what I and my family eat.
VF7	I prefer to take dietary supplements rather than attach greater importance to eating fully balanced meals.
VF8	Taste is more important to me than the energy value of the food I consume.
VF9	Since having a child/children, I attach more importance to the quality of the food I consume.
VF10	It is important to me that the quality of the food products I buy is appropriate to the price I pay.

**Table 3:** The results of U Mann–Whitney (U) and Kruskal–Wallis test (H).

Variables		SP		FP		EF		VF	
		Mean (Std)	U or H_(DoF)_ Statistic	Mean (Std)	U or H_(DoF)_ Statistic	Mean (Std)	U or H_(DoF)_ Statistic	Mean (Std)	U or H_(DoF)_ Statistic
Sex									
	Women	22.7 (2.93)	ns	25 (3.94)	ns	36.22 (7.14)	ns	36.11 (4.52)	ns
	Men	22.74 (3.39)		23.81 (3.81)		34.78 (7.69)		34.78 (3.4)	
Age									
	Less than 23	21.75 (2.14)	ns	25.44 (4.62)	ns	37.56 (8.17)	ns	34.47 (3.38)	U = 175.5 *p* = 0.015 r = 0.33
	23 and more	23.13 (3.42)		23.97 (3.51)		34.63 (6.96)		37.75 (4.57)	
Education									
	Elementary	23.85 (1.77)	ns	22.69 (2.32) *	H_(2, 54)_ = 6.94 *p* = 0.031 η^2^ = 0.097	31.85 (4.85) *	H_(2, 54)_ = 7.72 *p* = 0.021 η^2^ = 0.11	33.31 (2.98) *	H_(2, 54)_ = 8.67 *p* = 0.013 η^2^ = 0.13
	Secondary	22.7 (3.86)		24.37 (3.64)		34.78 (7)		35.26 (3.75)	
	Higher	21.71 (2.27)		26.07 (4.92) *		40.29 (7.97) *		37.79 (4.35) *	
Employment									
	Student	23.29 (3.36)	U = 163 *p* = 0.008 r = −0.36	24.03 (3.58)	ns	34.61 (7.08)	ns	34.74 (3.64)	ns
	Employed	21.38 (2.06)		25.31 (4.54)		37.63 (7.88)		37.13 (4.49)	
No. of household members									
	1 to 3	22.82 (2.59)	ns	24.86 (4.21)	ns	37.59 (7.46)	ns	36.59 (4.06)	ns
	4 and more	22.66 (3.51)		24.09 (3.68)		34.06 (7.09)		34.66 (3.86)	
Voivodship									
	Silesian	23.15 (3.16)	ns	23.92 (4.29)	ns	36.31 (7.58)	ns	36.23 (4.17)	ns
	Lower Silesian	23.26 (4.04)		24.68 (3.97)		34 (6.94)		34.37 (3.7)	
	Lesser Poland	22 (2.07)		24.45 (3.73)		36.32 (7.77)		35.91 (4.17)	

Legend: ns, not significant; U, results of Mann–Whitney U test when comparing two groups, H—results of Kruskal–Wallis test when comparing more than two groups, SP, Food product selection, FP, Food preparation and production, EF, Methods of food consumption, VF, Views on food and nutrition, *—indicates significant post-hoc between elementary and higher education levels.

## Data Availability

Data will be available on request from the corresponding author.
